# A novel mechanism for C1GALT1 in the regulation of gastric cancer progression

**DOI:** 10.1186/s13578-021-00678-2

**Published:** 2021-08-26

**Authors:** Xiaoxia Dong, Chunli Chen, Xinzhou Deng, Yongyu Liu, Qiwen Duan, Zhen Peng, Zhiguo Luo, Li Shen

**Affiliations:** 1 Department of Clinical Oncology, Taihe Hospital, Hubei University of Medicine, Shiyan, 442000 Hubei China; 2 School of Basic Medical Sciences, Hubei University of Medicine, Shiyan, 442000 Hubei China

**Keywords:** Gastric cancer, Malignant progression, Glycosyltransferase, C1GALT1

## Abstract

**Background:**

Gastric cancer (GC) is a highly aggressive and lethal disease around the world. High expression of core 1 β 1, 3-galactosyltransferase 1 (*C1GALT1*), the primary enzyme responsible for protein *O*-glycosylation, plays a critical role in gastric carcinogenesis. However, proteins that can be *O*-glycosylated by C1GALT1 in GC have not been completely elucidated. Also, the mechanism leading to its upregulation in GC is currently unknown.

**Results:**

Using public databases and our patient samples, we confirmed that C1GALT1 expression was upregulated at both the mRNA and protein levels in GC tissues. Elevated expression of C1GALT1 protein was closely associated with advanced TNM stage, lymph node metastasis, tumor recurrence, and poor overall survival. With gain- and loss-of-function approaches, we demonstrated that C1GALT1 promoted GC cell proliferation, migration, and invasion. By employing lectin pull-down assay and mass spectrometry, integrin α5 was identified as a new downstream target of C1GALT1 in GC. C1GALT1 was able to modify *O*-linked glycosylation on integrin α5 and thereby modulate the activation of the PI3K/AKT pathway. Functional experiments indicated that integrin α5 inhibition could reverse C1GALT1-mediated tumor growth and metastasis both in vitro and in vivo. Moreover, transcription factor SP1 was found to bind to the C1GALT1 promoter region and activated its expression. Further investigation proved that miR-152 negatively regulated C1GALT1 expression by directly binding to its 3′ -UTR.

**Conclusions:**

Our findings uncover a novel mechanism for C1GALT1 in the regulation of GC progression. Thus, C1GALT1 may serve as a promising target for the diagnosis and treatment of GC.

**Supplementary Information:**

The online version contains supplementary material available at 10.1186/s13578-021-00678-2.

## Background

Gastric cancer (GC) is one of the most frequently occurring malignancies in China and worldwide [1, 2]. Despite improvements in diagnostic and therapeutic strategies, the prognosis of GC patients remains very poor [3]. The high mortality among GC patients is partially due to its high invasive and metastatic ability [4]. Hence, further insight into the mechanisms underlying GC occurrence and progression is becoming increasingly important.

Glycosylation is a ubiquitous and important post-translational modification. Most of the proteins can be glycosylated with *N*-linked (attached to Asn) or *O*-linked (attached to Ser or Thr) glycans. As a hallmark of cancers, abnormal glycosylation leads to the formation of tumor-associated glycans or glycoproteins [5, 6]. Glycosylation is mainly controlled by the actions of glycosyltransferases, and altered expression of glycosyltransferases results in the production of specific glycans on proteins [7]. Until now, about 300 glycosyltransferases have been identified in the Carbohydrate Active enzyme database. Glycosyltransferases and alterations in protein glycosylation have crucial functions in various pathological processes [8–10]. Core 1 β 1, 3-galactosyltransferase 1 (*C1GALT1*) is the only enzyme that is responsible for the biosynthesis of core 1 *O*-glycans (Galβ1-3GalNAcα1-Ser/Thr). C1GALT1 controls the crucial step of GalNAc-type *O*-glycosylation and is a key contributor to human oncogenesis [11–14]. A recent study reported that C1GALT1 expression was elevated in GC and correlated with gastric carcinogenesis [15]. However, proteins that can be *O*-glycosylated by C1GALT1 in GC have not been completely elucidated. Also, the factors that contribute to its upregulation in GC are currently unknown.

The present study aimed to discover the regulatory mechanisms of C1GALT1 in GC progression. Our results may help to develop novel prognostic biomarkers and potential therapeutic targets for GC.

## Results

### C1GALT1 is overexpressed in GC and predicts poor prognosis

**Fig. 1:**
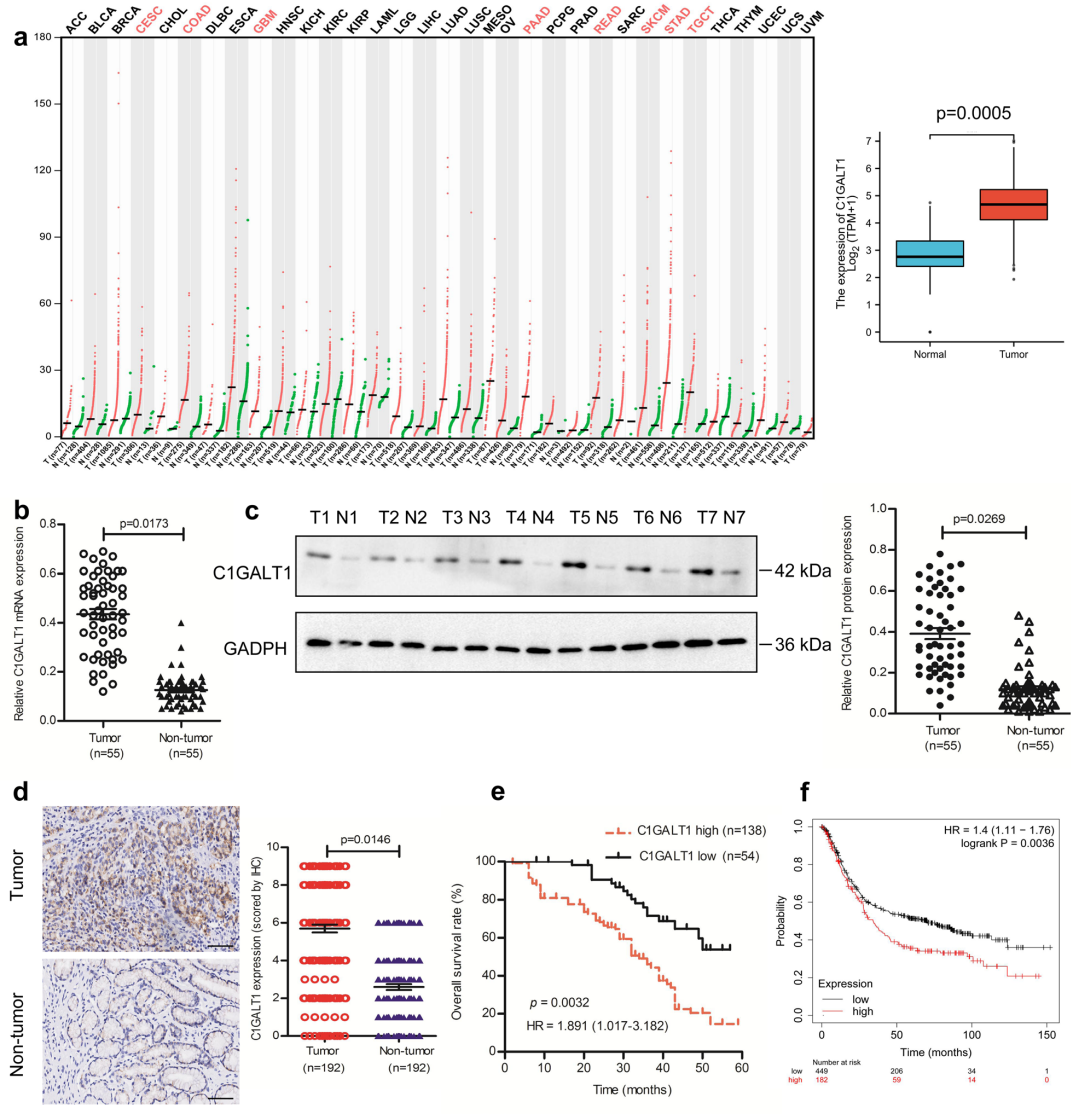
C1GALT1 is upregulated in GC tissues and high expression of C1GALT1 predicts poor prognosis for GC patients. **a** Bioinformatics analysis of *C1GALT1* mRNA expression in 33 types of cancers using the TCGA and GTEx databases (*p* = 0.005, Mann–Whitney U test). **b** qRT-PCR analysis of *C1GALT1* expression in paired GC tissues and their adjacent non-tumor tissues (*p* = 0.0173, Mann–Whitney U test). **c** Western blot analysis of C1GALT1 expression in paired GC tissues and their adjacent non-tumor tissues (*p* = 0.0269, Mann–Whitney U test). **d** Representative images and quantitative analysis of C1GALT1 staining. Scale bars, 100 μm (*p* = 0.0146, Mann–Whitney U test). **e** Kaplan–Meier analysis of overall survival in GC patients according to C1GALT1 expression (*p* = 0.0032, Kaplan–Meier method with log-rank test). **f** Kaplan–Meier survival curves of C1GALT1 in GC patients generated from the Kaplan–Meier plotter (*p* = 0.0036, Kaplan–Meier method with log-rank test). STAD: stomach adenocarcinoma; T: tumor tissue; N: normal or non-tumor tissue

To get an overall profile of C1GALT1 expression in different cancers, we analyzed the expression levels of *C1GALT1* mRNA in 33 types of cancers using RNA-sequencing data derived from the Cancer Genome Atlas (TCGA) and Genotype-Tissue Expression (GTEx) databases. It showed that *C1GALT1* mRNA was overexpressed in a variety of tumors, including GC (Fig. 1a). Then qRT-PCR was performed using fresh-frozen tissues to validate the expression of *C1GALT1* mRNA in GC. We found that *C1GALT1* mRNA in GC tissues displayed higher levels than in matched adjacent non-tumor tissues (Fig. 1b). The overexpression of C1GALT1 in GC tissues was confirmed at the protein level by Western blot and IHC (Fig. 1c, d). C1GALT1 expression was significantly associated with TNM stage, lymph node metastasis, and tumor recurrence (Table 1). Survival analysis suggested that high C1GALT1 expression was remarkably correlated with reduced overall survival of GC patients (Fig. 1e). Consistently, an online Kaplan–Meier plotter indicated that GC patients with high expression of C1GALT1 had shorter overall survival (Fig. 1f). Univariate analysis identified four factors associated with prognosis: TNM stage, lymph node metastasis, tumor recurrence, and C1GALT1 expression. Multivariate analysis revealed that C1GALT1 was an independent unfavorable prognostic factor (Table 2). These results highlight the importance of C1GALT1 for GC tumorigenesis and prognosis.

**Table 1:** Association between C1GALT1 expression and clinicopathological parameters in GC

Variables	Number of cases	C1GALT1		P-value
		Low (54)	High (138)	
Age (years)				
< 60	60	15	45	0.072
≥ 60	132	39	93	
Gender				
Male	116	30	86	0.351
Female	76	24	52	
Tumor size (cm)				
< 5	68	18	50	0.533
≥ 5	124	36	88	
Differentiation				
Well, moderately	51	10	41	0.196
Poorly	141	44	97	
TNM stage				
I + II	87	37	50	0.039*
III + IV	105	17	88	
Grade				
1–2	82	23	59	0.062
3–4	110	31	79	
Recurrence				
Absent	88	32	46	0.011*
Present	104	22	92	
Lymph node metastasis				
Negative	93	45	48	0.005*
Positive	99	9	90	

*p < 0.05

**Table 2:** Univariate and multivariate analysis for GC patients using the Cox regression model

Variables	Univariate analysis		Multivariate analysis	
	HR (95% CI)	P-value	HR (95% CI)	P-value
Gender Male vs. Female	1.068 (0.729–1.602)	0.831	1.302 (0.746–2.267)	0.324
Age (years) < 60 vs. ≥ 60	0.725 (0.446–1.189)	0.603	0.876 (0.535–1.434)	0.775
Tumor size (cm) < 5 vs. ≥ 5	1.039 (0.534–1.713)	0.292	0.914 (0.455–1.842)	0.802
Differentiation Well and moderately vs. Poorly	1.577 (1.048–1.959)	0.304	1.539 (0.799–2.966)	0.181
TNM stage I + II vs. III + IV	2.533 (1.529–4.265)	0.009*	2.871 (1.506–5.393)	0.063
Grade 1–2 vs. 3–4	1.352 (0.887–2.008)	0.158	1.438 (0.779–2.702)	0.261
Recurrence Absent vs. Present	2.728 (1.695–4.013)	0.025*	2.305 (0.922–5.771)	0.052
Lymph node metastasis Negative vs. Positive	2.076 (1.243–3.022)	0.027*	1.356 (0.709–2.174)	0.033*
C1GALT1 expression Low vs. High	1.655 (1.102–2.474)	0.001*	1.773 (1.094–2.565)	0.004*

*p < 0.05

### C1GALT1 promotes the proliferation, migration, and invasion of GC cells

**Fig. 2:**
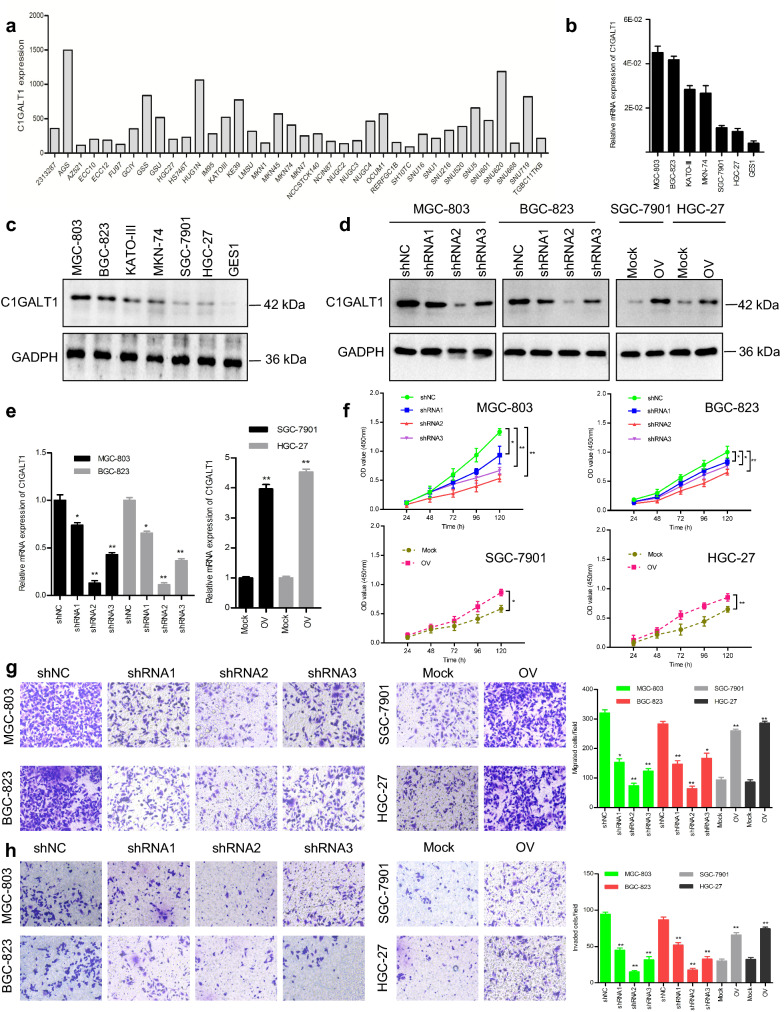
C1GALT1 promotes GC cell proliferation, migration, and invasion. **a** Bioinformatics analysis of *C1GALT1* mRNA expression in 38 GC cell lines using the CCLE database. **b** qRT-PCR analysis of *C1GALT1* expression in one normal gastric cell line and six GC cell lines. **c** Western blot analysis of C1GALT1 expression in different cell lines. **d** C1GALT1 protein expression was detected by Western blot after transfection. **e** *C1GALT1* mRNA expression was analyzed by qRT-PCR after transfection. **f** CCK-8 assay was used for proliferation analysis. **g** Transwell chambers without Matrigel were used for migration analysis. **h** Matrigel-coated Transwell chambers were used for invasion analysis. shNC: cells transfected with negative control lentivirus; shRNAs: cells transfected with *C1GALT1* shRNA lentivirus; Mock: cells transfected with empty plasmid; OV: cells transfected with *C1GALT1* plasmid. *p < 0.05, **p < 0.01 compared with the shNC or Mock group (Student’s t-test or one-way ANOVA)

To elucidate the functional role of C1GALT1 in GC, the expression profile data of 38 GC cell lines from the Cancer Cell Line Encyclopedia (CCLE) website were downloaded. The results showed that *C1GALT1* mRNA was constitutively expressed in all GC cell lines, but at very different levels (Fig. 2a). Meanwhile, qRT-PCR and Western blot analysis of C1GALT1 expression in one normal gastric cell line and six GC cell lines revealed that C1GALT1 was more frequently overexpressed in GC cell lines than that in normal cell lines (Fig. 2b, c). The relatively higher levels of C1GALT1 were found in MGC-803 and BGC-823 cells, whereas SGC-7901 and HGC-27 cells expressed lower levels. Thus, MGC-803 and BGC-823 cells were utilized for loss-of-function experiments, whereas SGC-7901 and HGC27 cells were employed for gain-of-function experiments (Fig. 2d, e). CCK-8, Transwell migration, and Matrigel invasion assays showed that overexpression or knockdown of C1GALT1 elevated or suppressed the proliferation, migration, and invasion of GC cells, respectively (Fig. 2f–h). These findings further confirm that C1GALT1 contributes to GC growth and metastasis, which is consistent with a previous report using other GC cell lines [15].

### Integrin α5 is a target glycoprotein of C1GALT1 in GC

**Fig. 3:**
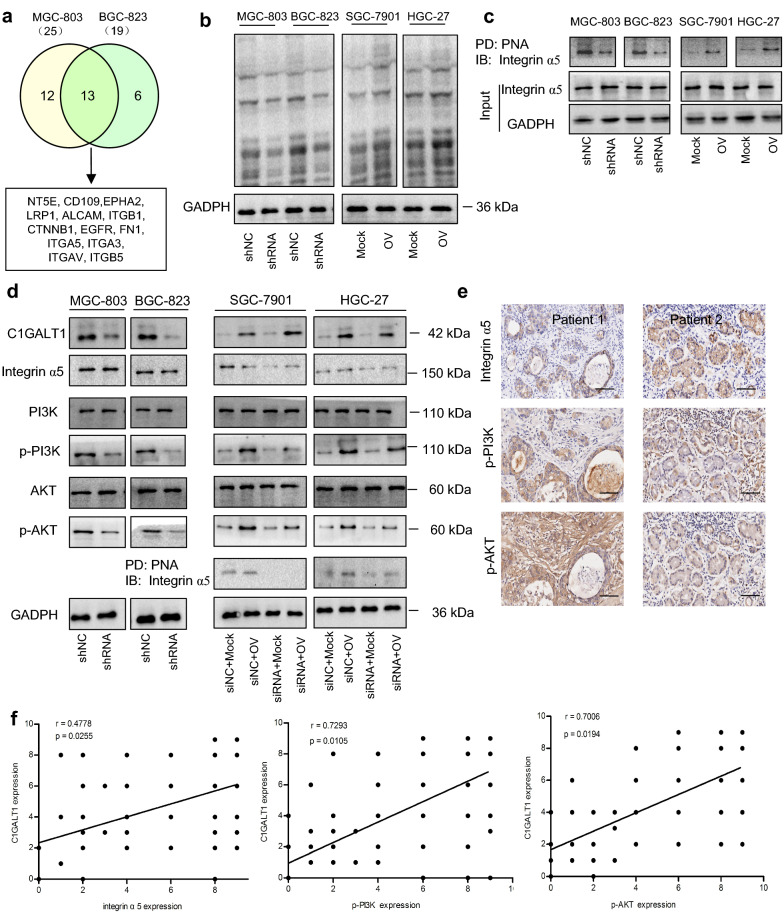
C1GALT1 regulates the *O*-glycosylation of integrin α5 in GC. **a** The number of PNA-binding proteins identified by mass spectrometry was displayed using Venny. **b** The cell-surface proteins that interacted with PNA were detected using a lectin-based pull-down assay. Membrane proteins were divided into two parts, one for GADPH detection, and the other for PNA-binding measurement. GAPDH was an internal control. **c** The proteins from whole-cell lysates enriched by PNA were analyzed by immunoblotting with an anti-integrin α5 antibody. **d** The indicated protein expression was evaluated by Western blot after transfection. **e** Representative images of integrin α5, p-PI3K, and p-AKT staining in GC samples. Scale bars, 100 μm. **f** Correlation analysis (Spearman’s rank correlation test). shNC: cells transfected with negative control lentivirus; shRNA: cells transfected with *C1GALT1* shRNA2 lentivirus; Mock: cells transfected with empty plasmid; OV: cells transfected with *C1GALT1* plasmid; siNC: cells transfected with negative control siRNA; siRNA: cells transfected with integrin α5 siRNA; IB: immunoblot; PD: pull-down

To identify the downstream effectors of C1GALT1 in GC, the membrane extracts from MGC-803 and BGC-823 cells were prepared and *O*-glycosylated proteins were enriched using lectin PNA. After proteomic analysis and data filtration, 25 and 19 potential PNA-binding proteins were identified in MGC-803 and BGC-823 cells, respectively, 13 of which were common to both cell lines (Fig. 3a, Additional file 1: Table S1). Notably, integrin α5 could be detected in both cell lines. Integrin α5 has been revealed to be involved in cancer development and progression [16–18]. Bioinformatics analysis indicated that integrin α5 was closely related to GC, but its relationship with C1GALT1 has not yet been reported [19, 20]. Therefore, integrin α5 was selected as a candidate to study. Next, we examined the glycosylation state of integrin α5. We found that overexpression or knockdown of C1GALT1 increased or decreased the binding of PNA to cell-surface and integrin α5 (Fig. 3b, c). Meanwhile, C1GALT1 had no obvious effect on the protein expression of integrin α5. Thus, the interaction between C1GALT1 and integrin α5 is bridged by *O*-glycosylation.

Integrin α5 is an upstream regulator of the PI3K/AKT pathway [19]. We then investigated whether C1GALT1 was associated with the regulation of integrin α5/PI3K/AKT axis in GC cells. Western blot analysis showed that the C1GALT1 knockdown was able to inhibit the activation of the PI3K/AKT pathway (Fig. 3d). Moreover, PI3K/AKT pathway could be activated by C1GALT1 overexpression, and this tendency was blocked by integrin α5 inhibition. Besides, correlation studies in GC tissues demonstrated that C1GALT1 was weakly correlated with integrin α5 but strongly correlated with p-PI3K and p-AKT (Fig. 3e, f). These results suggest that C1GALT1 activates PI3K/AKT pathway in GC, which may be dependent on *O*-glycosylation of integrin α5.

### Integrin α5 is a key mediator in C1GALT1-induced GC growth and metastasis

**Fig. 4:**
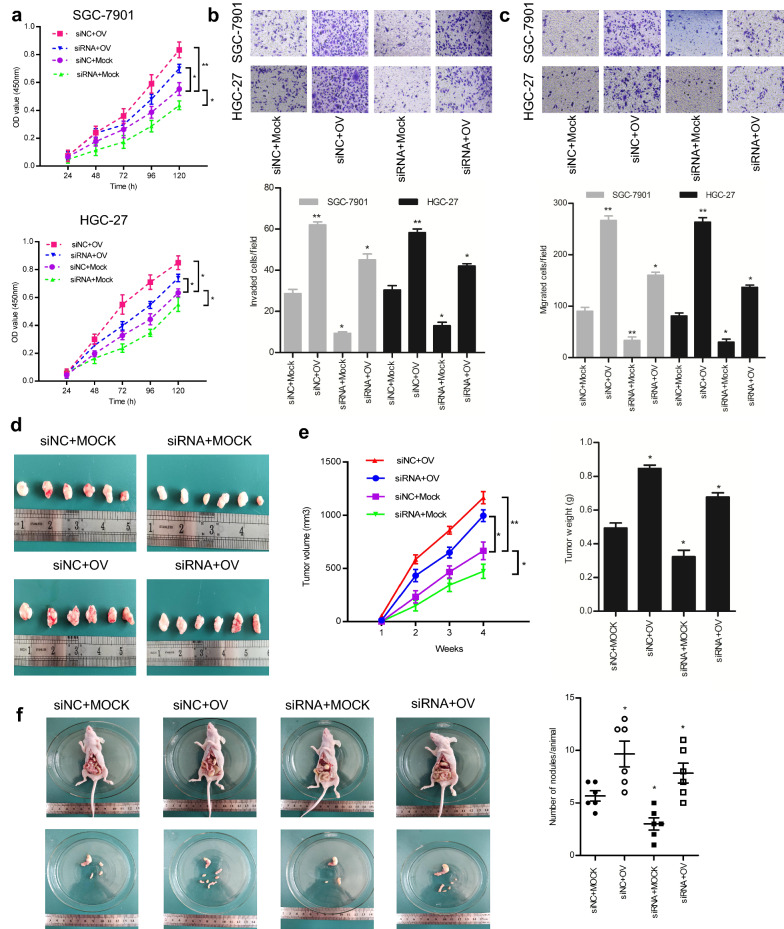
Effects of C1GALT1 on GC proliferation, migration, and invasion are mediated by integrin α5. **a–c** Cell proliferation, migration, and invasion were assessed by CCK-8, Transwell migration, and Matrigel invasion assays. **d** Photographs of xenograft tumors. **e** The tumor growth curve and tumor weight in the nude mice. **f** Metastatic nodules were photographed and counted. n = 6 mice per group. Mock: cells transfected with empty plasmid; OV: cells transfected with *C1GALT1* plasmid; siNC: cells transfected with negative control siRNA; siRNA: cells transfected with integrin α5 siRNA. *p < 0.05, **p < 0.01 compared with the siNC+Mock group (Student’s t-test or one-way ANOVA)

To explore the functional relevance of integrin α5 in C1GALT1-mediated GC progression, CCK-8, Transwell migration, and Matrigel invasion assays were performed. We found that inhibition of integrin α5 attenuated the potentiation effects of C1GALT1 overexpression on GC cell proliferation, migration, and invasion (Fig. 4a–c). To validate our in vitro results, we established a subcutaneous tumor model and a peritoneal metastatic xenograft model in nude mice, respectively. The results showed that the promotive effects of C1GALT1 overexpression on tumor growth were weakened by integrin α5 inhibition (Fig. 4d, e). Furthermore, C1GALT1 overexpression led to a significant increase in the number of visible peritoneal nodules. In contrast, integrin α5 inhibition reduced the increased visible peritoneal nodules induced by C1GALT1 (Fig. 4f). Overall, our data support integrin α5 as a functionally important target protein of C1GALT1 in GC.

### SP1 transcriptionally upregulates C1GALT1 expression in GC

**Fig. 5:**
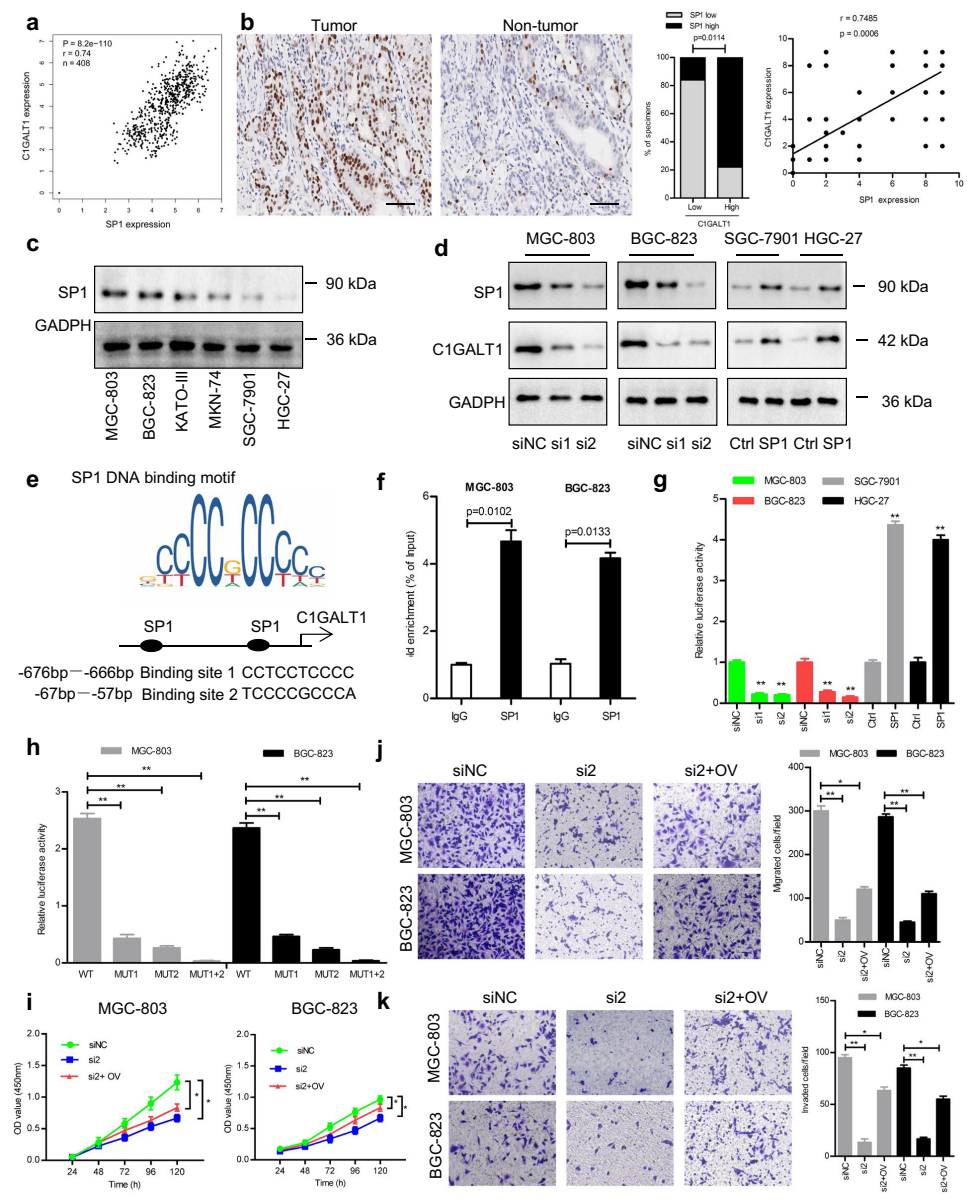
C1GALT1 is transcriptionally activated by SP1 in GC. **a** Scatter diagrams for C1GALT1 expression versus SP1 expression in GC samples based on the TCGA and GTEx databases(Spearman’s rank correlation test). **b** Representative images and quantitative analysis of SP1 expression in GC tissues (Spearman’s rank correlation test). Scale bars, 100 μm. **c** Western blot analysis of SP1 expression in different GC cells. **d** The effect of SP1 on C1GALT1 expression was detected by Western blot. **e** Schematic presentation of SP1 binding sites within the promoter region of *C1GALT1*. **f** The binding of SP1 to the *C1GALT1* promoter was analyzed by ChIP-PCR. **g**, **h** Relative luciferase activity was examined by the dual-luciferase reporter assay. **i**–**k** Cell proliferation, migration, and invasion were assessed by CCK-8, Transwell migration, and Matrigel invasion assays. siNC: cells transfected with negative control siRNA; si1: cells transfected with SP1 siRNA1; si2, cells transfected with SP1 siRNA2; Ctrl, cells transfected with control plasmid; SP1, cells transfected with SP1 plasmid; OV: cells transfected with *C1GALT1* plasmid; WT: Wild-type; MUT: mutant. *p < 0.05, **p < 0.01 compared with the siNC or Ctrl group (Student’s t-test or one-way ANOVA)

Transcription factors are essential for modulating gene expression. The 9 top-ranked transcription factors predicted by the GeneCards platform were ATF-2, c-Jun, GATA-1, HNF-1 A, IKZF2, NFE2L1, MAZR, PPAR-α, and SP1. We performed a correlation analysis between these transcription factors and C1GALT1 expression in GC using the TCGA and GTEx datasets. A strong association was observed between C1GALT1 and SP1 but not other transcription factors (Fig. 5a, Additional file 1: Figure S1). IHC analysis of GC tissues showed that SP1 expression was positively correlated with C1GALT1 expression (Fig. 5b). The expression of SP1 in different GC cells exhibited a consistent tendency with that of C1GALT1 (Fig. 5c). Furthermore, C1GALT1 expression was decreased after SP1 knockdown but increased after SP1 overexpression (Fig. 5d, Additional file 1: Figure S2). Considering a specific role for SP1 in the control of C1GALT1 transcription, we next analyzed the promoter region of C1GALT1 via the JASPAR database. Through bioinformatics analysis, two potential SP1 binding sites (-676/-666; -67/-57) were identified (Fig. 5e). ChIP enrichment analysis indicated that SP1 could bind the *C1GALT1* promoter region (Fig. 5f). The dual-luciferase reporter assays revealed that *C1GALT1* promoter activity was enhanced or inhibited when SP1 was overexpressed or knocked down, respectively (Fig. 5g). We also found that mutation of either binding site reduced promoter activity, whereas mutation of both binding sites resulted in a complete loss of activity (Fig. 5h). We further discovered that SP1 knockdown inhibited cell proliferation, migration, and invasion. However, these inhibitory effects were reversed by C1GALT1 overexpression (Fig. 5i–k). Collectively, these results imply that SP1 is required for regulating C1GALT1 expression in GC.

### Decreased miR-152 contributes to C1GALT1 overexpression in GC

**Fig. 6:**
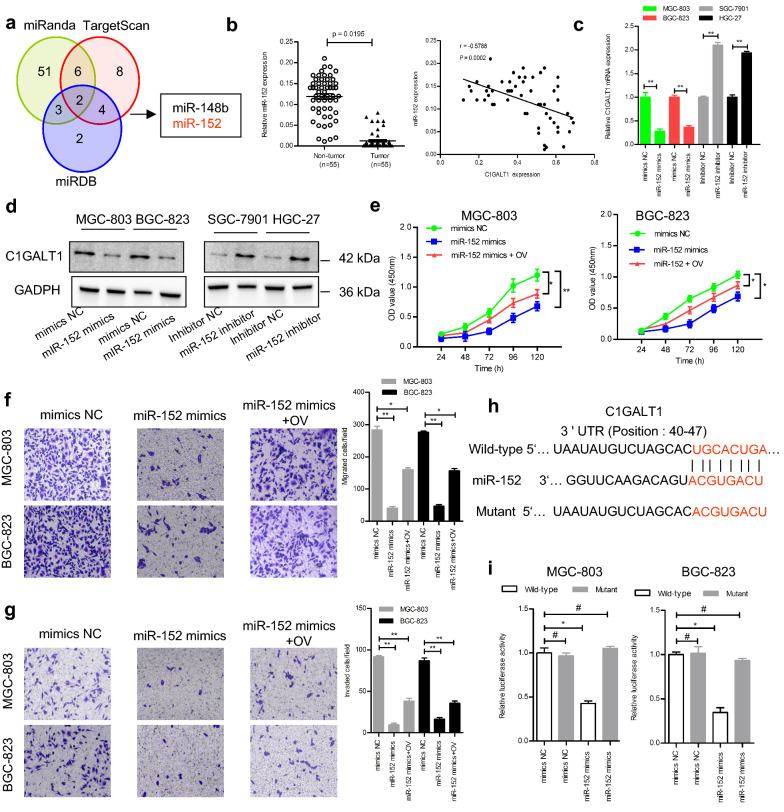
C1GALT1 is a direct target of miR-152 in GC. **a** Venn diagrams depicting the number of potential miRNAs targeting the 3ʹUTR of *C1GALT1*. **b** Correlation of miR-152 and C1GALT1 expression in GC tissues (Spearman’s rank correlation test). **c**, **d** qRT-PCR and Western blot analysis of C1GALT1 expression after miR-152 overexpression or inhibition. **e**–**g** Cell proliferation, migration, and invasion were assessed by CCK-8, Transwell migration, and Matrigel invasion assays. **h** Diagram of the potential binding sequences of miR-152 on the 3′-UTR of *C1GALT1* and the mutated sequences of *C1GALT1* 3′-UTR. **i** Relative luciferase activity was examined by the dual-luciferase reporter assay. OV: cells transfected with *C1GALT1* plasmid. *p < 0.05, **p < 0.01, ^#^*p* > 0.05 compared with the mimics NC or inhibitor NC group (Student’s t-test or one-way ANOVA)

MiRNAs are generally considered to post-transcriptionally and negatively regulate gene expression. To explore whether the expression of C1GALT1 was regulated by specific miRNAs, three bioinformatics software (TargetScan, miRanda, and miRDB) were utilized to predict the miRNAs that can bind to 3ʹ-UTR of *C1GALT1* mRNA. We found that C1GALT1 was a predicted target of miR-148b and miR-152 (Fig. 6a). Although qRT-PCR analysis revealed that these two miRNAs were down-regulated in GC tissues, only miR-152 was significantly negatively correlated with C1GALT1 expression (Fig. 6b, Additional file 1: Figure S3). C1GALT1 expression was suppressed by overexpression of miR-152 and increased by knockdown of miR-152 (Fig. 6c, d). Phenotypic experiments showed that miR-152 overexpression led to a prominent reduction in cell proliferation, migration, and invasion. Meanwhile, these effects could be reversed by the restoration of C1GALT1 (Fig. 6e–g). Sequence analysis demonstrated that the 3′-UTR of *C1GALT1* mRNA had complementary binding sites with miR-152 (Fig. 6h). Moreover, miR-152 overexpression repressed the luciferase activity of the wild-type but not the mutant 3ʹ-UTR of C1GALT1 (Fig. 6i). Thus, miR-152 functions as a negative regulator of C1GALT1 in GC.

## Discussion

**Fig. 7:**
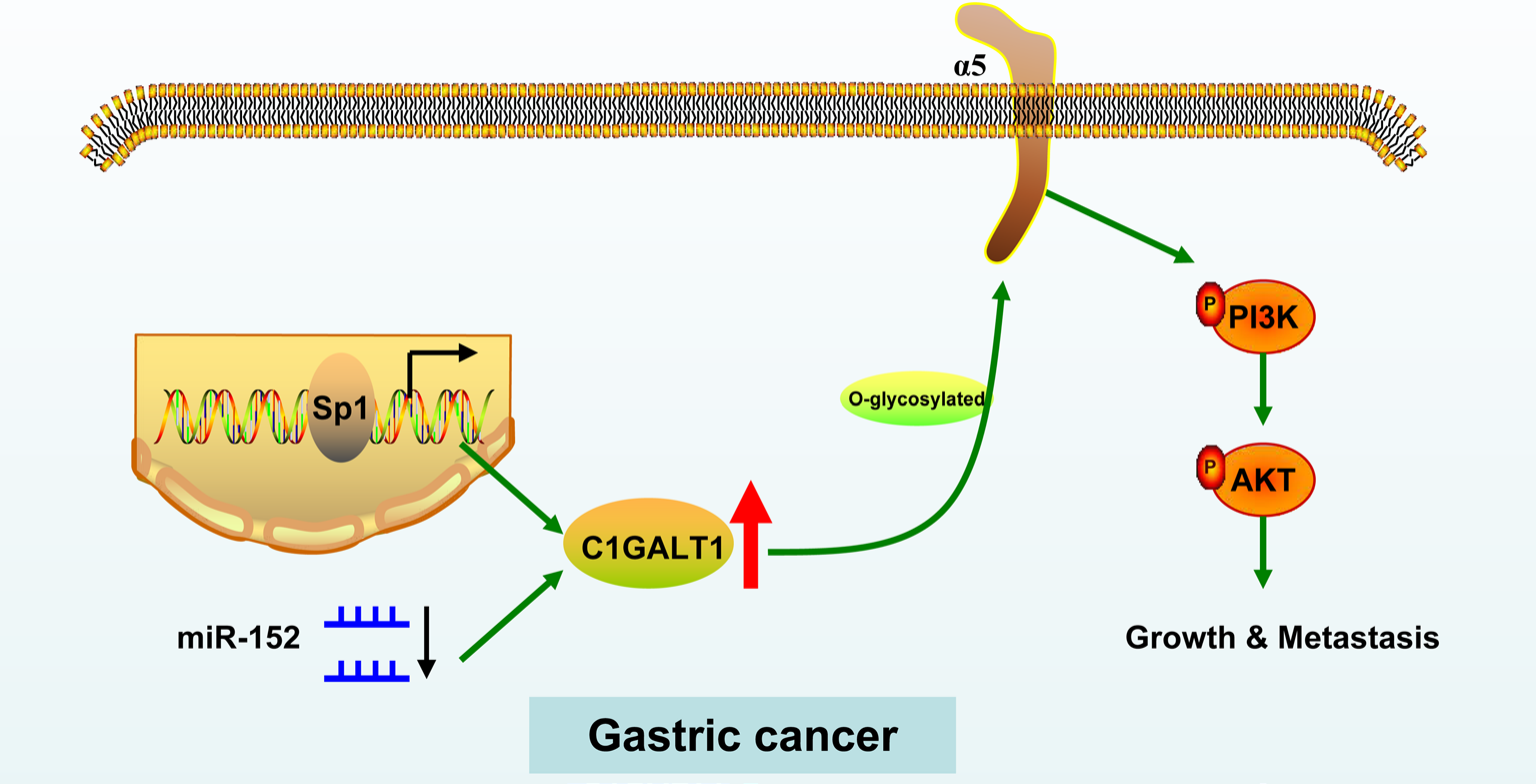
Diagram of the proposed mechanism showing how C1GALT1 modulates GC progression

The present study investigated the clinical significance and biological function of C1GALT1 in GC. The downstream effectors (*O*-glycosylated proteins) and upstream regulators (transcription factors and miRNAs) of C1GALT1 in GC were also explored. C1GALT1 was found to be overexpressed in GC and was a marker of poor prognosis. C1GALT1 played a central role in the malignant progression of GC via modifying integrin α5 *O*-glycosylation and activating the PI3K/AKT pathway. Moreover, C1GALT1 was transcriptionally upregulated by SP1, and decreased miR-152 further contributed to *C1GALT1* mRNA stability in GC (Fig. 7). Our findings uncover a novel mechanism for C1GALT1 in promoting GC progression. To our knowledge, this is the first study to explore the detailed mechanism of C1GALT1 upregulation in GC. The regulatory relationship between C1GALT1 and integrin α5 was also firstly reported in GC.

Altered expression of glycosyltransferases is crucial in determining the development, progression, and aggressiveness of GC [21–23]. A previous study analyzed the association between C1GALT1 expression and clinical characteristics of 98 GC patients by IHC [15]. The current study analyzed a series of public databases, including TCGA, GTEx, and the Kaplan–Meier plotter, to get an overall expression profile and prognostic value of C1GALT1 in GC. Besides, qRT-PCR, Western blot, and IHC were performed in 192 GC samples to verify the findings obtained from the bioinformatics analysis. Using different vectors, cell types, and animal models, we further provide evidence indicating that C1GALT1 can function as an important contributor to GC growth and metastasis.

The downstream events following C1GALT1 dysregulation are linked to the alteration in protein *O*-glycosylation. For example, knockout of C1GALT1 led to the truncation of *O*-glycosylation on MUC16 in pancreatic adenocarcinoma [24]. *O*-glycosylation of EGFR was blocked by C1GALT1 knockdown in head and neck cancer [11]. C1GALT1 modified the *O*-glycosylation of MET in hepatocellular carcinoma [25]. These studies also confirmed that altered *O*-glycosylation was closely associated with tumor proliferation, invasion, and metastasis. In this study, we discovered that integrin α5 was a potential substrate for C1GALT1 during GC progression. Integrins are a family of transmembrane glycoprotein receptors, which contain both *N*- and *O*-linked glycosylation sites [26]. Integrin α5 *N*-glycosylation plays a crucial role in multiple biological processes, including cell adhesion and migration [27, 28]. However, the mechanism of integrin α5 *O*-glycosylation in GC remains unclear. Using lectin pull-down assay, we found that C1GALT1 overexpression increased, while knockdown decreased, the *O*-glycosylation of integrin α5. Functional experiments indicated that integrin α5 inhibition could reverse C1GALT1-mediated tumor growth and metastasis both in vitro and in vivo. Thus, integrin α5 is a key mediator in C1GALT1-induced GC progression. In addition to integrin α5, our studies also supply many other glycoproteins that still need to be explored.

So far, the underlying mechanism accounting for C1GALT1 upregulation in GC remains not completely understood. SP1 is an essential transcription factor for gene regulation and can drive the expression of many cancer-related genes. It is widely reported that abnormal expression of SP1 contributed to GC tumorigenesis [4, 29, 30]. In the present study, paralleled expression of C1GALT1 and SP1 in GC was observed. SP1 was a positive regulator of C1GALT1 via binding to its promoter. miR-152 is a type of abnormally expressed miRNAs in many types of malignancies including GC [31–33]. We found that the expression of miR-152 was negatively correlated with that of C1GALT1 in GC. miR-152 served as a negative regulator of C1GALT1 via direct interaction with its 3ʹ-UTR. Despite the large number of target genes of SP1 and miR-152 have been identified, the relationship between these two molecules and C1GALT1 is still unknown. Our study provides the first experimental evidence that SP1 and miR-152 act in concert to promote GC growth and metastasis by regulating C1GALT1.

There are several limitations to our study. For instance, the number of tissue specimens that we have studied is not large, although substantial. We have shown the presence of integrin α5 in GC tissues. Unfortunately, we are unable to confirm the expression of *O*-glycosylated integrin α5 in these samples. In addition, it is currently unclear which sites on integrin α5 are *O*-glycosylated by C1GALT1. Moreover, the mechanism of synergy between SP1 and miR-152 requires further investigation. It would be of interest to investigate whether these findings can be extended to other tumors.

## Conclusions

In summary, we reported that increased C1GALT1 expression was an effective predictor of worse prognosis in GC. C1GALT1 potentiated GC growth and metastasis by targeting integrin α5. *C1GALT1* was transcriptionally activated by SP1 and was post-transcriptionally controlled by miR-152. Hence, C1GALT1 may serve as a novel oncogene during GC progression. Our study will provide an important insight into the pathogenesis of GC and supply potential targets for new drug inventions.

## Methods

### Patients samples and cell lines

The Ethics Committee of Hubei University of Medicine (Shiyan, China) approved all research. Archived GC tissue specimens were collected from patients undergoing surgery in Taihe Hospital without radio- or chemotherapy after obtaining written informed consent. The samples were divided into two parts, one was fixed in formalin and another was stored at -80 °C. GC cell lines (KATO-III, BGC-823, MGC-803, SGC-7901, MKN-74, HGC-27) and gastric mucosal epithelial cell line (GES-1) were purchased from Procell (Wuhan, China). Cells were cultured in RPMI-1640 or Hams F12 medium (Gibco, USA) containing 10% fetal bovine serum (FBS; Gibco).

### Immunohistochemistry (IHC) staining

IHC was conducted as previously described [34]. The staining degree was calculated as follows: Overall score = intensity score (0, negative; 1, weak; 2, moderate; and 3, strong) × percentage score (0, 0–5%; 1, 6–25%; 2, 26–50%; 3, 51–75%; 4, ≥ 76%). A final score ≥ 4 was considered as high expression and < 4 as low expression. The following antibodies were used: C1GALT1 (ab237734, 1:500, Abcam, USA), integrin α5 (ab150361, 1:100, Abcam), p-PI3K (abs103557, 1:200, Absin, China), p-AKT (abs130889, 1:150, Absin), and SP1 (ab124804, 1:150, Abcam).

### Oligonucleotide and plasmid transfection

*C1GALT1* shRNA lentivirus plasmid, *C1GALT1* overexpression plasmid, integrin α5 siRNAs, SP1 siRNAs, SP1 overexpression plasmid, miR-152-3p mimics, miR-152-3p inhibitor, and their corresponding negative controls were acquired from GenePharma (Suzhou, China). The sequences for shRNAs and siRNAs were listed in Additional file 1: Table S2. Cell transfection was performed using Lipofectamine 3000 (Invitrogen, USA) or siRNA-mate (GenePharma). Transfection efficiency was evaluated by quantitative real-time PCR (qRT-PCR) and Western blot after transfection. Stable cell lines were selected with puromycin (Sigma, USA).

### qRT-PCR and western blot

Total RNA was reverse-transcribed into cDNA using TransScript First-Strand cDNA Synthesis SuperMix (TransGen, China). qRT-PCR reactions were conducted using SYBR PCR Master Mix (ABI, USA). The primers were listed in Additional file 1: Table S3. The relative gene expression was normalized to control using the 2^−ΔΔCt^ method. Total protein was quantified using the BCA protein assay kit (Biosharp, China). The approach for Western blot was conducted as described previously [34]. The following antibodies were used: C1GALT1 (ab237734, Abcam), integrin α5 (ab52971, Abcam), PI3K (4255, CST, USA), p-PI3K (4228, CST), AKT (9272, CST), p-AKT (9271, CST), SP1 (ab124804, Abcam), and GADPH (BL006B, Biosharp).

### Cell proliferation, migration, and invasion assays

Cells (2 × 10^3^/well) were seeded into 96-well plates and cell viability was examined at 24, 48, 72, and 96 h using Cell Counting Kit-8 (CCK-8; Dojindo, Japan). The migratory and invasive abilities were measured using 8-µm transwell chambers (24-well insert, Corning, USA) coated with (invasion assay) or without (migration assay) Matrigel (BD Biosciences, USA), respectively. 3 × 10^4^ cells in serum-free medium were seeded in the upper chamber. After 24 h (migration assay) or 36 h (invasion assay), cells on the lower side of the chamber were stained and photographed.

### Lectin pull-down assay and mass spectrometry analysis

Membrane protein was extracted using a CelLytic MEM Protein Extraction kit (Sigma) and then incubated overnight with peanut agglutinin (PNA)-coated agarose beads (Vector Labs, USA) at 4 °C. The pulled-down proteins were subjected to 10% SDS-PAGE. For mass spectrometry analysis, the gels were stained with coomassie brilliant blue. Mass spectrometry was carried out based on the method as already described by us and others [8, 35]. For analysis of the modifications to cell-surface glycoproteins, proteins in the gels were electrophoretically transferred to a PVDF membrane (Millipore, USA). The membrane was probed with biotin-labeled PNA (Vector Labs). Subsequently, bands were visualized using HRP-conjugated streptavidin (Vector Labs). To evaluate the glycosylation status of integrin α5, total proteins from whole-cell lysates were prepared. After incubation with PNA-coated beads, the precipitated complexes were separated by SDS-PAGE and immunoblotted with the antibody against integrin α5.

### Chromatin immunoprecipitation (ChIP) assay

ChIP was performed using the EZ-ChIP™ Kit (Millipore, USA) [36]. DNA fragments were generated by ultrasound, followed by incubation with an anti-SP1 antibody (ab231778, Abcam) or IgG isotype antibody (ab172730, Abcam). DNA recovered from reverse cross-linking was used for qRT-PCR. The primers were listed in Additional file 1: Table S3.

### Luciferase reporter assay

The *C1GALT1* wild-type or mutant 3′-UTR was constructed and cloned into a pGL3 luciferase reporter plasmid (Promega, USA). Then the vectors combined with miR-152 mimics or NC were co-transfected into cells. Alternatively, *C1GALT1* promoter-luciferase reporter plasmid or binding-site mutant plasmid was transfected into cells along with the control plasmid. The luciferase activity was measured at 48 h after transfection using the Dual-Luciferase Reporter Assay System (Promega).

### Animal experiments

BALB/c nude mice (4-week-old, female) were obtained from the Animal Center of Hubei University of Medicine. Animal experiments were approved by the Animal Health Committee of the Hubei University of Medicine. To assess the tumor growth, 1 × 10^7^ SGC-7901 cells were subcutaneously injected into each mouse. After 4 weeks, mice were killed, and tumors were removed and weighed. To establish the peritoneal metastasis model, 2 × 10^6^ SGC-7901 cells were injected intraperitoneally into each mice. The mice were killed 4 weeks later, and the metastatic nodules were counted.

### Statistical analysis

Data were expressed as the mean ± SD. Differential gene expression analysis was performed using the Mann–Whitney U test. The student’s t-test or one-way analysis of variance was used for comparison between two groups or more than two groups, respectively. Spearman coefficient was used to analyze correlations. A strong correlation was assumed for 0.7 < |r| ≤ 1, a moderate correlation for 0.5 < |r| ≤ 0.7, a weak correlation for 0.3 < |r| ≤ 0.5, and no correlation for |r| ≤ 0.3. Pathologic parameters were analyzed using the χ2 test. The log-rank test was used for Kaplan–Meier survival analysis. A P-value < 0.05 was considered statistically significant.

## Supplementary Information

**Additional file 1: Figure S1.** Scatter diagrams for C1GALT1 expression versus transcription factors expression in GC samples based on the TCGA and GTEx databases. **Figure S2.** Effect of SP1 on C1GALT1 expression. *C1GALT1* mRNA levels were detected by qRT-PCR. siNC, cells transfected with negative control siRNA; si1, cells transfected with SP1 siRNA1; si2, cells transfected with SP1 siRNA2; Ctrl, cells transfected with control plasmid; SP1, cells transfected with SP1 plasmid. ^*^p < 0.05, ^**^p < 0.01 compared with the siNC or Ctrl group (Student’s t-test). **Figure S3.** Correlation of miR-148b and C1GALT1 expression in GC tissues (Spearman’s rank correlation test). **Table S1.** PNA-binding proteins identified by the proteomic analysis. **Table S2.** The sequence of shRNAs and siRNAs. **Table S3.** Primer sequences used in the qRT-PCR analysis.

## Data Availability

All data generated during this study are included in this published article and supplementary information files. Further details are available from the corresponding author upon request.

## Abbreviations

GC: Gastric cancer
*C1GALT1*: Core 1 β 1, 3-galactosyltransferase 1
IHC: Immunohistochemistry
qRT-PCR: Quantitative real-time PCR
ChIP: Chromatin immunoprecipitation
PNA: Peanut agglutinin
TCGA: The Cancer Genome Atlas
GTEx: Genotype-Tissue Expression
shRNA: Short hairpin RNA
siRNA: Small interfering RNA
UTR: Untranslated region
CCK-8: Cell Counting Kit-8
