# Supplementary material for: A novel mechanism for C1GALT1 in the regulation of gastric cancer progression
Source: Cell Biosci. 2021 Aug 26;11:166. doi: 10.1186/s13578-021-00678-2 (PMC8393437; doi:10.1186/s13578-021-00678-2)
Supplement: Supplementary file 1 — Additional file 1: Figure S1. Scatter diagrams for C1GALT1 expression versus transcription factors expression in GC samples based on the TCGA and GTEx databases. Figure S2. Effect of SP1 on C1GALT1 expression. C1GALT1 mRNA levels were detected by qRT-PCR. siNC, cells transfected with negative control siRNA; si1, cells transfected with SP1 siRNA1; si2, cells transfected with SP1 siRNA2; Ctrl, cells transfected with control plasmid; SP1, cells transfected with SP1 plasmid. *p < 0.05, **p < 0.01 compared with the siNC or Ctrl group (Student’s t-test). Figure S3. Correlation of miR-148b and C1GALT1 expression in GC tissues (Spearman’s rank correlation test). Table S1. PNA-binding proteins identified by the proteomic analysis. Table S2. The sequence of shRNAs and siRNAs. Table S3. Primer sequences used in the qRT-PCR analysis. [file 13578_2021_678_MOESM1_ESM.doc]

**

**

**Fig. S1** Scatter diagrams for C1GALT1 expression versus transcription factors expression in GC samples based on the TCGA and GTEx databases.

**
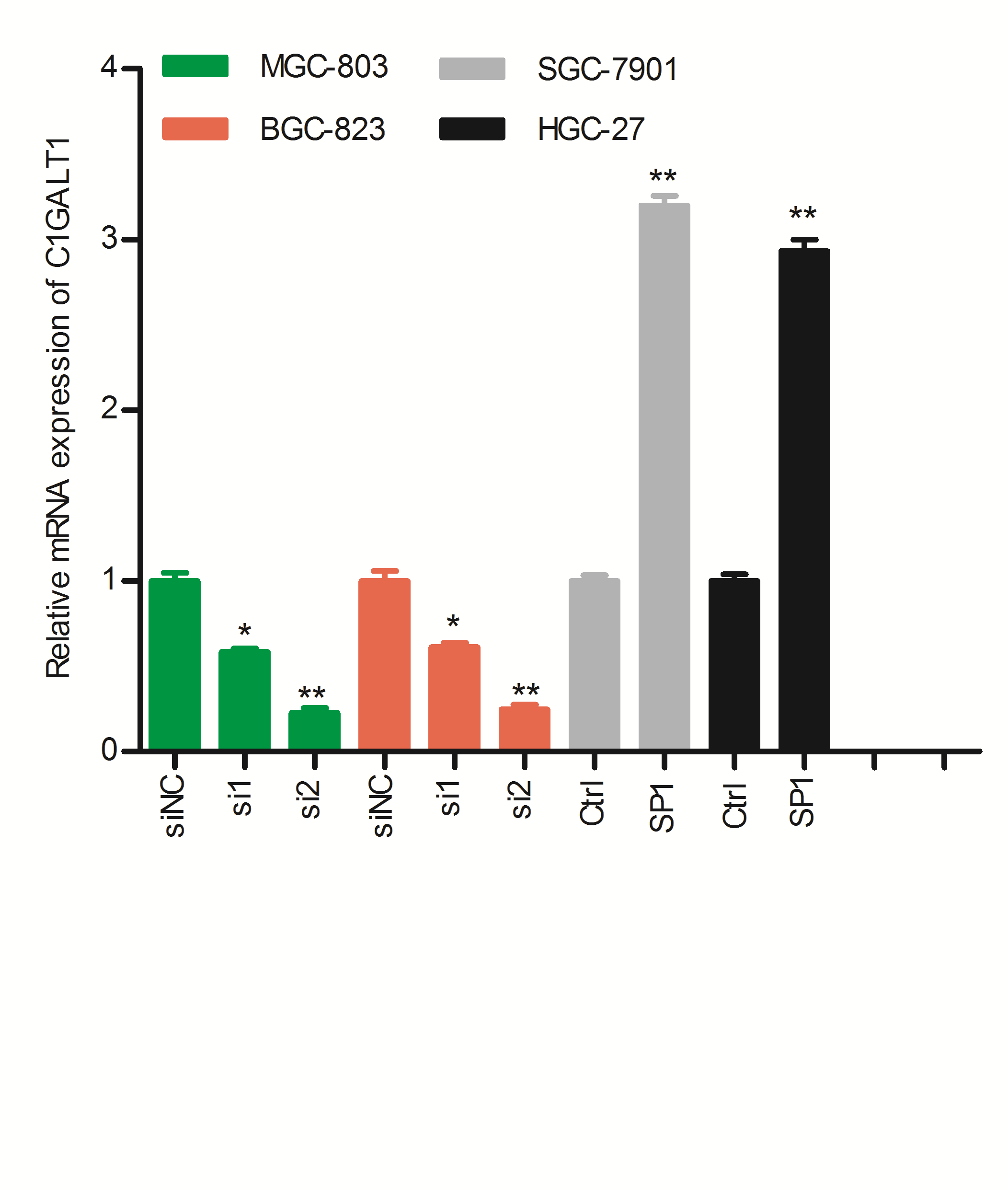
**

**Fig.S2** Effect of SP1 on C1GALT1 expression. *C1GALT1* mRNA levels were detected by qRT-PCR. sc, cells transfected with negative siRNA; si1, cells transfected with SP1 siRNA1; si2, cells transfected with SP1 siRNA2; Ctrl, cells transfected with control plasmid; SP1, cells transfected with SP1 overexpression plasmid. ^*^*p* < 0.05, ^**^*p* < 0.01 compared with the siNC or Ctrl group (Student’s t-test).

**
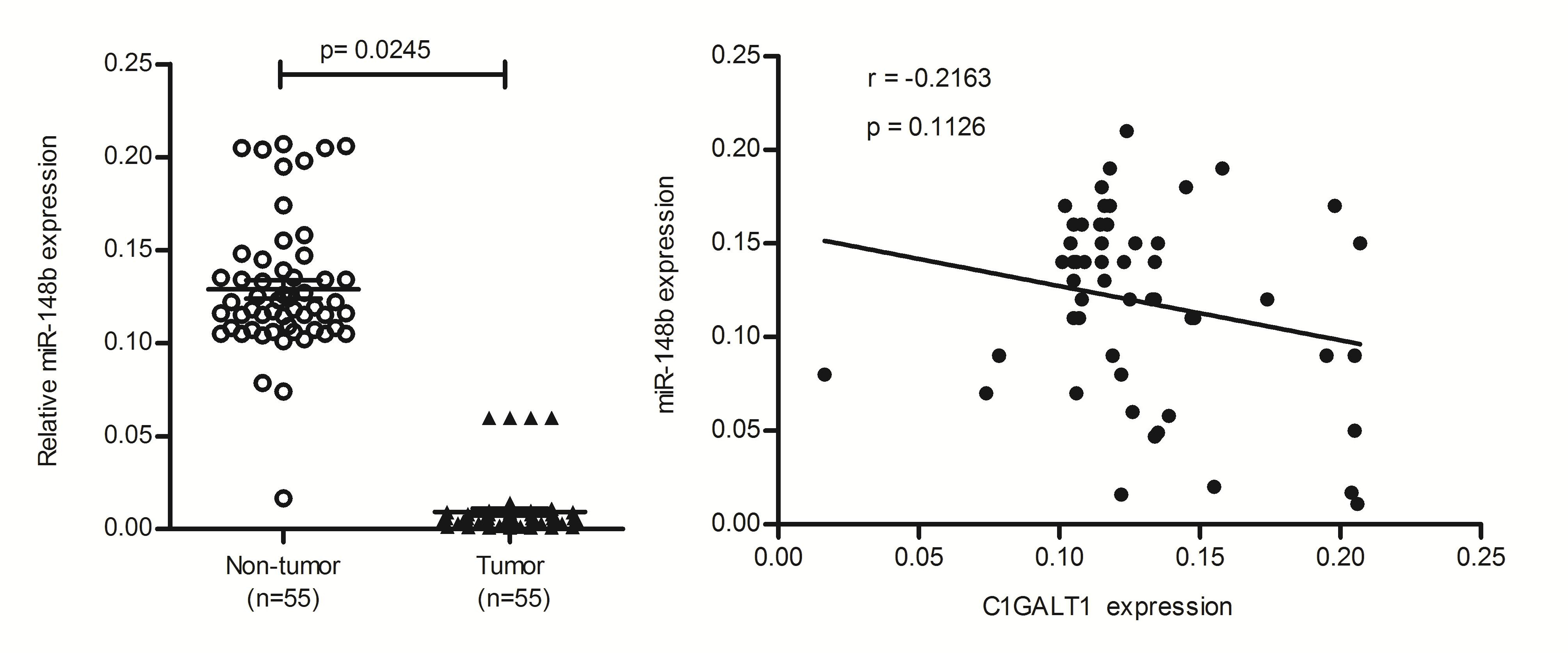
**

**Fig. S3** Correlation of miR-148b-3p and C1GALT1 expression in GC tissues (Spearman’s rank correlation test).

**Table S1.** PNA-binding proteins identified by the proteomic analysis

| **Cell type** | **Protein name** |
| --- | --- |
| AGS | 5'-nucleotidase ecto(NT5E),CD109 molecule(CD109), EPH receptor A2(EPHA2), LDL receptor related protein 1(LRP1), NPC intracellular cholesterol transporter 1(NPC1), activated leukocyte cell adhesion molecule(ALCAM), catenin β1(CTNNB1), collagen type VII α1 chain(COL7A1), epidermal growth factor receptor(EGFR), fibronectin 1(FN1), insulin like growth factor 1 receptor (IGF1R), **integrin subunit α5(ITGA5)**, integrin subunit α3(ITGA3), integrin subunit αV(ITGAV), integrin subunit β1 (ITGB1), integrin subunit β3(ITGB3), integrin subunit β5(ITGB5), laminin subunit β1(LAMB1), neuronal cell adhesion molecule(NRCAM), neuropilin 2(NRP2), pappalysin 1(PAPPA) plexin B2(PLXNB2), ribophorin I(RPN1), ribophorin II(RPN2), transmembrane protein 2(TMEM2), vimentin(VIM) |
| MGC-803 | 5'-nucleotidase ecto(NT5E), CD109 molecule(CD109), EPH receptor A2(EPHA2), ER lipid raft associated 2(ERLIN2), LDL receptor related protein 1(LRP1), activated leukocyte cell adhesion molecule(ALCAM), cadherin 2(CDH2), catenin β 1 (CTNNB1), desmoglein 2(DSG2), epidermal growth factor receptor(EGFR), fibronectin 1(FN1), insulin like growth factor 2 receptor (IGF2R), **integrin subunit α 5(ITGA5)**, integrin subunit α 3(ITGA3), integrin subunit αV(ITGAV), integrin subunit β 1(ITGB1), integrin subunit β 3(ITGB3), integrin subunit β 5(ITGB5), leucyl and cystinyl aminopeptidase(LNPEP) |

**Table S2.** The sequence of shRNAs and siRNAs

| **Name** | **Sequences (5**′**-3**′**)** |
| --- | --- |
| C1GALT1 shRNA1 | GCAAGGCATTCAGATGATAAT |
| C1GALT1 shRNA2 | GGTTGACACCCAGCCTAATGT |
| C1GALT1 shRNA3 | GCCAACATAAAGATGAGAACA |
| NC shRNA | TTCTCCGAACGTGTCACGT |
| integrin α5 siRNA | GCAUUCAAUCCAGGAGAAATT |
| SP1 siRNA 1 | CCAAGGAAAUAAGGACAGUCUAGCT |
| SP1 siRNA 2 | CCCUCAACCCUAUUCAUUAGCAUTA |
| NC siRNA | UUCUCCGAACGUGUCACGUTT |

**Table S3.** Primer sequences used in the qRT-PCR analysis

| **Name** |  | **Sequences (5′-3′)** |
| --- | --- | --- |
| C1GALT1 | Forward  Reverse | TAGAGGGTCCTGGTTGCTGCT CAGTGCAGTGCTAGACATATTAC |
| SP1 | Forward  Reverse | GACGTTGATGCCACTGTTGGCAAG  TCAAGACCCACCAGAATAAGAAGGGAG |
| miR-152 | Forward  Reverse | ACACTCCAGCTGGGTCAGTGCATGACAG  CTCAACTGGTGTCGTGGAGTCGGCAATTCA |
| U6 | Forward  Reverse | CTCGCTTCGGCAGCACA  AACGCTTCACGAATTTGCGT |
| GADPH | Forward  Reverse | GGACCTGACCTGCCGTCTAG  GTAGCCCAGGATGCCCTTGA |
| C1GALT1-CHIP-PCR | Forward  Reverse | AAGCATCCCCTTTCTCGCTC  CTAATGCGAAGGGGTCTGGG |
